# Drivers of Nutritional Change in Pakistan: A Decomposition Analysis

**DOI:** 10.3390/nu15143124

**Published:** 2023-07-13

**Authors:** Azka Rehman, Ping Qing, Xia Cui

**Affiliations:** 1 School of Economics and Statistics, Guangzhou University, Guangzhou 510006, China; 2 College of Economics and Management, Huazhong Agricultural University, Wuhan 430070, China; qingping@mail.hzau.edu.cn

**Keywords:** child undernutrition, decomposition, stunting, underweight, child health, Pakistan

## Abstract

The global reduction in child undernutrition highlights the international and national commitment to prioritizing future generations’ health. This study aims to find out the previous trends in nutrition and the key drivers of these changes in different regions of Pakistan. For empirical investigation, we employed a regression-based decomposition model by using two distinct rounds of demographic and health surveys: 2012-13 and 2017-18. Results showed children with stunted growth reduced substantially in Balochistan, while Punjab exhibited the highest progress for underweight children. Regression estimates showed that maternal nutritional status and household wealth were more pronounced in reducing all measures of child malnutrition. Some determinants, including mother age at marriage and prenatal visits to the hospital, are significant only for long-term nutritional status—stunting, while mother education contributed to reducing wasted and underweight children. The rest of the factors, like father education and household environment, appear to play a humble role in explaining nutritional improvements. From disaggregated analysis based on different regions, we found that modeled factors used in this study explained changes disproportionately. Thus, it is recommended to use multidimensional nutrition policies incorporating a broader range of sectors, and region-specific programs should be designed for more effective outcomes.

## 1. Introduction

Child undernutrition can have immediate as well as long-run consequences for the mental and physical development of the child [1]. Preliminary empirical studies and meta-analyses have accentuated that the negative effects of suboptimal child growth, measured as stunted growth, spread around many areas [2]. Firstly, poor linear growth affects the cognitive, emotional, social, and physical development of the child, which makes them vulnerable to illness, disability, and premature death [3,4]. Secondly, a child with stunted growth has serious consequences for their socio-economic outcomes in adulthood in the form of poor educational attainment, employment, and earnings [5]. One of the studies from Pakistan finds that children who have poor growth are more likely to have lower primary school enrollment [6].

It is well understood that the economic situation at the national level helps to deal with health problems given better infrastructure, while at the household level it gives more access to food and health resources. But we have witnessed many countries progressing economically faster than their improvement in health indicators. The possible explanation is that there are some underlying determinants at the individual level other than income, which plays a significant role in child health production. Existing academic literature guided us to single out key determinants responsible for child malnutrition, ranging from child and parental characteristics to household environment. The most widely discussed determinants at the parental level are their education, maternal age at marriage, nutritional status, and access to health facilities. Furthermore, household wealth status and a nourishing environment in the form of access to clean drinking water and the availability of hygienic sanitation also ensured better nutritional growth [7,8,9,10].

For a long time, child undernutrition had been surging, but recently we have started seeing downward trends in many Asian and African countries, including Pakistan. Researchers observed that the recent progress in child health globally can be attributed to various determinants that vary across different parts of the world. The contribution of household wealth is prominent in Bangladesh, Nepal, and India but relatively modest in Zambia, and health care access is favorable toward children living in Senegal. Parental education was found to be a key indicator in most countries except Zambia, where bed nets contributed the most [11]. Moreover, the success of China and Vietnam can be attributed to agricultural-leg strategies; Brazil’s food security is achieved by social protection programs; and Thailand’s success is driven by both strategies. Considering the variation in results across different countries motivated this study since there is no existing study focusing on drivers of reduction in undernutrition in Pakistan.

To understand the relative contribution of each factor to burgeoning child nutrition status in Pakistan, we incorporated the regression-based decomposition method proposed by Oaxaca and Blinder [12] by using sample datasets collected by the demographic health survey. Previously, the decomposition method was mostly employed in discrimination literature but recently caught the attention of researchers to investigate changes over time. Similar to other econometric techniques, this approach also faces criticism; some academicians argue that since it is based on partial equilibrium theory, it cannot provide detailed analysis [13]. But it merited consideration on the basis of providing exploratory detail on relationships between different variables before conducting more costly experimental research. This analytical approach is particularly relevant for our analysis because our primary objective is to gain an initial understanding of the essential factors that drive nutritional change over time. In addition to the aggregated country analysis, we have also conducted a separate analysis for each region, keeping in mind the diversity within a country, which may lead to distinct factors of significance in different regions.

## 2. Data and Estimation

Data for this study came from the most recent two rounds of the Pakistan demographic and health survey, PDHS 2012-13 and PDHS 2017-18. It is a nationally representative cross-sectional dataset carefully designed to collect data on hypothesized factors of child health and nutrition, thus being well suited to meet the objectives of this study. The PDHS 2012-13 survey collected data from 14,000 households from 500 primary sampling units through a multistage stratified sampling procedure. Similarly, a representative sample of 16,240 households was selected from 580 primary sampling units. For this study, we further restricted the sample to ever-married women of childbearing age (15–49 years) who at least have one child below age 5, which left us with a 2857 sample size from PDHS 2012-13 and a 3582 sample size from PDHS 2017-18. The data was collected from four provinces of Pakistan: Punjab, Sindh, Khyber Pakhtunkhwa, and Balochistan, and two regions: Gilgit Baltistan and Islamabad Capital Territory (ICT). We excluded two areas, Azad Jammu and Kashmir and FATA, since no data were available in the 2012-13 survey.

### 2.1. Dependent Variable

Child nutrition is measured by three anthropometric measures: height-for-age (HAZ), weight-for-height (WHZ), and weight-for-age (WAZ). The conversion of all indicators to *z*-scores was performed by following the World Health Organization’s (WHO) worldwide standards of normal child growth. In addition to continuous variables, a binary variable for each indicator was also introduced: stunting, wasting, and underweight. A child is considered stunted if the height-for-age *z*-score is less than −2 standard deviation from the median of the WHO international growth reference for the same age and sex. A child with less height for their age shows continuation of insufficient nutrition intake and poor health for longer period of time. Wasting is defined as weight-for-height below −2 standard deviation and suggest rapid and severe weight loss. A child whose weight-for-height *z*-score is less than −2 standard deviation is categorized as underweight; it is a combination of HAZ and WHZ, meaning a child may have stunted growth, wasted growth, or both. Children are divided on the basis of binary coding of “1” if stunted, wasted, or underweight, or “0” otherwise.

### 2.2. Independent Variable

The explanatory variables were finalized after a critical review of existing studies; largely, the factors can be divided into child-level, parent-level, and household level variables. Three child characteristics were found to be influential in determining nutritional status: age, gender, and birth order. Child age in months was used as a continuous variable; gender was a binary variable code of “1” for females and “0” for males and birth order was also used as a continuous variable. At the parent level, we added mother age at marriage as a continuous variable while categorizing mother education into five categories: “0” for no education, “1” for primary, “2” for middle, “3” for secondary, and “4” for tertiary and above. Mother nutritional status was defined as body mass index (BMI) measured in kg/m^2^, divided into three categories: “0” underweight (less than 18.5), “1” normal (18.5–24.9), and “2” overweight (≥25). Access to health facilities was measured as antenatal visits to the hospital: “0” no visits; “1” less than 4 visits; “2” four or more visits. Mother’s employment was added as a binary variable coded “0” for not employed, “1” for employed, and finally the father’s education in years was added as a continuous variable.

At the household level, a binary variable was added for both the availability of safe drinking water and hygienic sanitation. To measure household wealth status, a detailed set of assets was used to calculate an index. Relative to income and consumption, this measure of wealth is less volatile and thus arguably a better long-run measure of household economic status. Since the existing wealth index calculated by DHS can only be used for a single point in time, we calculated a new index following the official guidelines of DHS.

At first, we pooled the data from both surveys on 41 dichotomous items measuring household ownership of assets and housing quality, along with two continuous variables (members per sleeping room and land area). We removed the hygienic sanitation and safe drinking water variables from the index based on their individual roles in health production functions. Then, by employing confirmatory factor analysis, two separate indices were constructed for households in urban and rural areas since different variables define wealth in each region. For example, in urban areas, there is a higher availability of publicly provided services, while in rural areas, households have more land and an animal herd. Finally, both indices were combined to form a single composite index by incorporating regression coefficients obtained from regressing the common score over urban and rural wealth scores. The resulting index was used as an independent variable in multivariate analysis. In addition to these variables; a binary variable for place of residence (urban “1” and rural “0”) and region the household belongs to were also added.

### 2.3. Econometric Specification

We use the Oaxaca–Blinder (OB) decomposition regression model to estimate the change in child nutritional indicators over the course of five years and identify the associative drivers of this change. The coefficient values obtained from the model provide the individual as well as collective contribution of all predictors to change in nutritional *z*-scores. The simple equation where we assume a linear dependent variable (in our case, HAZ, WHZ, and WAZ scores) is:

(1) $${\bar{Y}}^{2}-{\bar{Y}}^{1}=\left[\overset{k}{\underset{i=1}{\sum }}\beta_{i}^{2}\left({\bar{X}}_{i}^{2}-{\bar{X}}_{i}^{1}\right)\right]+[\overset{k}{\underset{i=1}{\sum }}{\bar{X}}_{i}^{1}\left(\beta_{i}^{2}-\beta_{i}^{1}\right)]$$

where ${\bar{X}}_{i}$ is vector of average values of explanatory variables and $\beta_{i}$ shows the coefficient estimates for survey year *i* (1 = 2012/2013, 2 = 2017/2018). The first term on the right-hand side of the equation represents compositional change attributable to a difference in characteristics, also referred to as the explained part of the model. The second part reflects component change identifiable as difference in coefficients or an unexplained part. However, we will only discuss the explained part of aggregation and the individual contribution of all covariates.

The use of the decomposition method is not straightforward for categorical explanatory variables, especially those without low to high degree values; this is known as the “identification problem”. The decomposition estimates would depend on the choice of reference (omitted) category in the model, which is not possible to fix with a simple equation. Yun [14] has proposed a practical solution for quantifying normalized effects. It is similar to averaging the coefficient effect of nominal variables while varying the reference groups. We used “normalize” in STATA with all regional binary variables instead of considering one province as a reference group for better estimation and interpretation of results.

Since the OB decomposition method is based on mean predicted values of dependent variables, for binary nutrition variables (stunting, wasting, and underweight), we used the Fairlie [15] extension of the OB model. One of the concerns with non-linear models is the order of variables inclusion in the process of decomposition, also known as “path dependency”. The Fairlie technique solves this issue with the random ordering of variables across multiple replications of decomposition. We used two options: “ro” for random ordering of the variable and “rep(1000)” for the number of replications (the results are not presented in the main text and can be provided on request). The decomposition for the non-linear equation can be expressed as:

(2) $${\bar{Y}}^{2}-{\bar{Y}}^{1}=\left[\;\frac{1}{N^{1}}\overset{N^{1}}{\underset{j=1}{\sum }}F\left(\beta^{2}X_{j}^{1}\right)-\frac{1}{N^{2}}\overset{N^{2}}{\underset{j=1}{\sum }}F\left(\beta^{2}X_{j}^{2}\right)\right]+\left[\;\frac{1}{N^{1}}\overset{N^{1}}{\underset{j=1}{\sum }}F\left(\beta^{1}X_{j}^{1}\right)-\frac{1}{N^{1}}\overset{N^{1}}{\underset{j=1}{\sum }}F\left(\beta^{2}X_{j}^{1}\right)\right]\;$$

where $N_{j}$ refer to the sample size of each survey year *j* (1 = 2012-13, 2 = 2017-18). We used clustered weights with the “svy” command for all analyses to overcome the under-representation problem of small geographical areas.

## 3. Results

### 3.1. Changes in the Child Nutritional Outcomes

**Figure 1:**
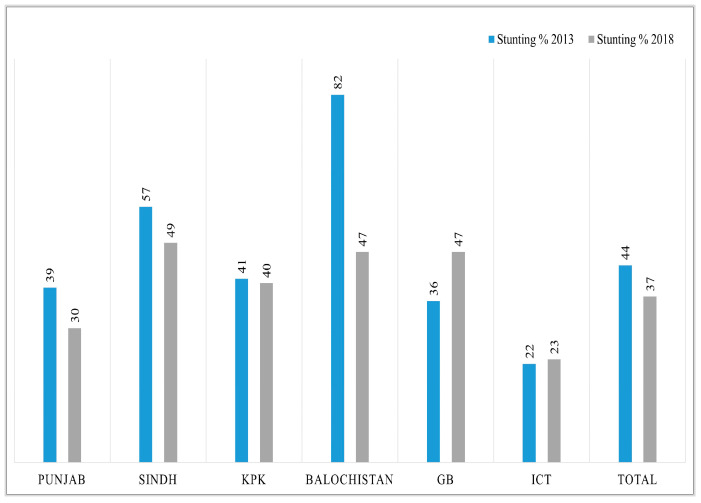
Percentage of stunted children in different regions of Pakistan.

**Figure 2:**
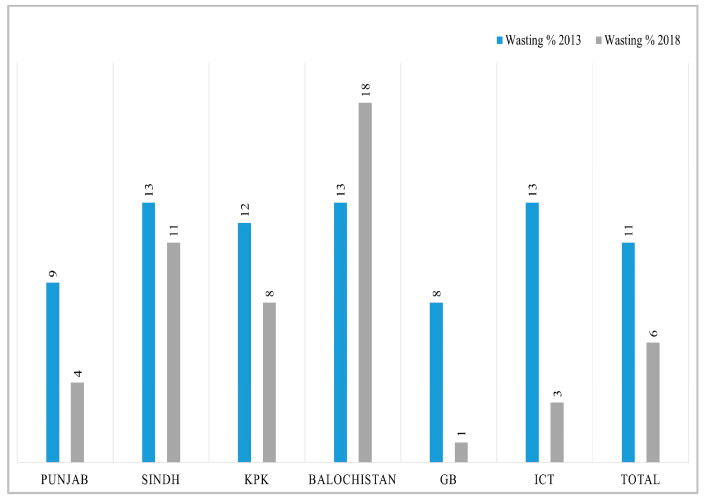
Percentage of wasted children in different regions of Pakistan.

**Figure 3:**
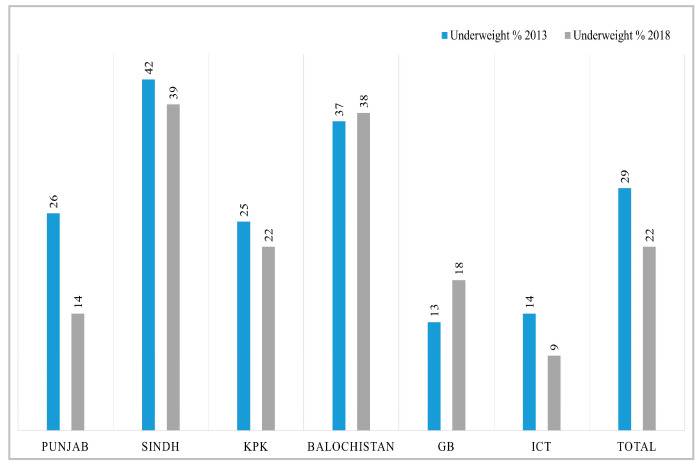
Percentage of underweight children in different regions of Pakistan.

**Figure 4:**
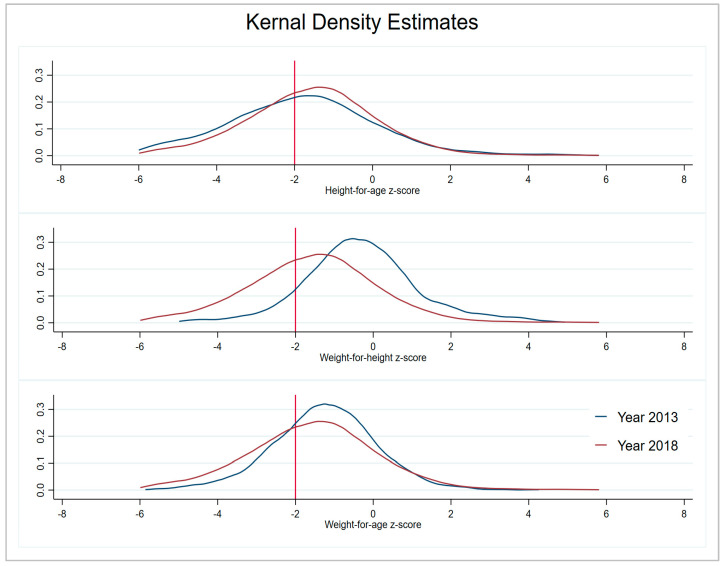
Kernel density estimates of child nutritional growth.

Before conducting decomposition analysis, we calculated simple descriptive changes in key variables from 2012-13 to 2017-18. The overall change in mean HAZ score is 0.23; for WHZ it is 0.26; and WAZ has increased by 0.28 in five years. More specifically, in each region, the highest increase in HAZ score has been observed in Balochistan with 1.99 points and the lowest in KPK 0.09, while the GB and ICT have shown a slight decrease. For the WHZ score, the highest improvement can be seen in ICT, and the lowest is in Sindh. The mean WAZ score has increased substantially in Punjab by 0.4 points; children living in Sindh showed a modest increase of 0.05 points, while GB exhibits a decline in *z*-score over time (Table 1). Similarly, the visualization of the difference in percentage of stunted, wasted, and underweight children between two time periods in different regions of Pakistan can be observed in Figure 1, Figure 2 and Figure 3. The kernel density estimates presented in Figure 4 also show that, over time, there has been a considerable improvement in all three measures of nutrition.

### 3.2. Changes in Key Drivers of Child Nutritional Growth

Table 2 depicts the changes in key determinants of child nutrition. It is widely documented that adolescent pregnancy carries the risk of children being born with less weight, which later translates into poor nutritional growth. Thus, delaying the mother’s age at marriage is a crucial factor in improving a child’s nutritional outcome [16]. We have observed a slight increase (one year) over time in women’s mean age at marriage. Another influential factor that can affect a child’s nutritional status is the mother’s own health. Our data showed that a woman’s BMI of less than 18.5 has decreased by four percent while that of women over 25 has increased by 11 percent in five years. By having good health, there are fewer chances of a low birth weight for the child, which is another crucial determinant of the child’s nutritional status. Mother’s proper nutrition also ensures a child’s initial nutritional requirement in the early 1000 days, which shapes a child’s mental and physical development in later stages of life [17]. However, similar to undernutrition, excess nutrition carries its own risks to mothers’ own health, including hypertension, diabetes mellitus, etc., and as a result also affects future generations’ linear growth (double burden of nutrition).

Prenatal visits to the hospital, which serve as a measure of access to healthcare, have been widely recognized as a key determinant of children’s weight and height in numerous countries [11]. We observed a significant 12 percent increase in the number of families seeking prenatal care at hospitals, with a visit frequency exceeding four times during pregnancy. This indicates a notable improvement in healthcare utilization among expecting families, reflecting a positive trend towards prioritizing maternal and child health. According to Aslam and Kingdon [18], the education level of mothers has consistently been identified as a key factor in improving child welfare through various channels. Notably, there has been a positive shift in educational attainment among women, with a four percent increase in those receiving tertiary education or above. Simultaneously, there has been an eight percent decrease in the proportion of women with no formal education. These trends highlight the significance of maternal education in promoting better outcomes for children.

Along with mother education, father education also improved by 1% in the past five years, which again ensures the general availability and access to food and health-related items in the household. Some studies have found that father education is also one way to combat the undernutrition crisis [19]. The combined effect of an educated father and mother may lead to a family with healthy children.

The economic status of a household is widely recognized as a highly debated and significant factor that guarantees the availability of ample nutrition required for a healthy life [20]. Sample data shows that the number of households that fall in the rich quantile increased by nine percent, and households with poor wealth reduced by nine percent, while households with middle wealth status remained the same. Not only household wealth determines child health, but its overall environment is also a requisite to defining the malnutrition status of its residents. The availability of safe water and hygienic sanitation facilities determines the utilization of available food. Unavailability of safe drinking water prevents the absorption of nutrients due to water-borne diseases [21]. Likewise, without proper sanitation facilities, it is hard to ensure that the food consumed is properly converting into the nutrients required for the body’s normal functioning. Our data show that in Pakistan, the situation of safe drinking water has slightly decreased by 1 percent; however, the number of families with safe sanitation facilities has improved by 12 percent over the past five years.

### 3.3. Decomposition Results

We initiated estimations with a simple ordinary least squares regression model and a logit model for the binary outcome variable to understand the relationship between child nutrition and its associated factors (results can be provided on request). Among all of the determinants, only the most significant ones were finally added to the decomposition regression analysis.

Results from the estimation of Equations (1) and (2) produced a detailed decomposition of changes in nutrition into endowment and coefficient in five years; however, we will only discuss the earlier one. Table 3 and Table 4 exhibit that the nutritional status of children below age five has improved in Pakistan from 2012-13 to 2017-18 and the change can largely be explained by the independent variables used in this study with few exceptions (for some regions, some unobservable characteristics drive the improvement in nutrition). Generally, we found that the linear model has more explanatory power than the nonlinear model; for instance, the surge in mean height-for-age *z*-score is 81 percent explained by the predictors of this study, while the logit model is explaining only 53 percent improvement with the same set of covariates. Similarly, a model with a mean weight-for-height *z*-score explains 20 percent more variation than estimation with a binary variable. The difference between linear and non-linear decomposition analysis for the predicted weight-for-age *z*-score is 15 percent, with the latter explaining less. The logic behind the difference can be explained by the fact that the mean *z*-score contains more information about changes at multiple cut points, while the binary variable only shows the difference at one point.

Before discussing each parameter for all models in detail, we would like to present a general idea of the relative significance of regressors in explaining the changes in child nutrition over time. With few contrasts, we found that mother’s education, her body mass index, her age at marriage, prenatal visits to the hospital, and household wealth status explained most of the improvement in the child’s nutritional score. Among these, the highest proportion is attributed to the mother’s body mass index and household wealth status for all three scores of child nutrition. We now turn to the details of each regressor modeled in Table 3. Starting with mother age at marriage, it shared 8 percent variation in HAZ with significance at 10 percent, and the coefficient value was positive but not significant for WHZ, and WAZ. Secondly, a considerable increase in HAZ, WHZ and WAZ was observed for children whose mothers have a better nutritional score (20, 12, and 17 percent, respectively), with all parameters being significant at 1 percent. Thirdly, mother education showed a humble contribution to improving child nutrition, and the coefficients were only significant for WHZ and WAZ (explaining 3 percent and 7 percent of the total difference, respectively).

At forth, the change in children HAZ whose mothers have visited health facilities at least four times before pregnancy was 21 percent with a 1 percent significant coefficient. Fifth, the father’s education is positive but not significant for either child nutrition measure. Sixth, the rise in the proportion of wealthy households considerably shifted children to HAZ (16 percent), WAZ (12 percent), and WAZ (8 percent). Finally, instead of using one provincial region as a reference, we added all provinces to the model and observed the changes in children’s nutritional scores in each region. The results highlighted that if we measure child nutrition as HAZ, the change is not significantly attributed to the residential area while keeping all other parameters constant. However, if we consider children’s weight-for-height *z*-score, children living in Punjab and GB are better off by 8 percent and 17 percent, respectively. Surprisingly, children’s weight-for-age *z*-score was negatively influenced if they lived in Balochistan and was better off with a residence in GB (more details on the individual contribution of parameters in each region are given in the next section).

In addition to the linear model, we also estimated non-linear equations, and the results are reported in Table 4. As expected, we found that the explained percentage of total change declined in aggregation as well as individually for all regressors. Some of the explanatory variables, like mother age at marriage, were reported to not contribute significantly to the probability of a child being stunted, while for wasted children, although all coefficients remained positive, they turned non-significant. The probability of children being underweight reduced over time, and the change is explained by 40 percent due to the mother’s own nutrition (13 percent), her education (10 percent), and the economic status of the household (13 percent), with a slight decrease in significance compared to the linearly estimated model.

#### 3.3.1. Regional Analysis

The descriptive statistics in the previous section highlighted that the progress in nutrition is inconsistent across different regions, and thus it would be wise to assume that the drivers of these changes may differ within a country. This led us to conduct separate decomposition analyses for each region to further enhance our understanding of geographical variation factors (see Supplementary File Tables S1–S3).

Punjab, being the most populous province in Pakistan, is assumed to play a substantial role in the country’s progress in nutrition. We found that the progress in height-for-age *z*-score is positive but not significant and has been driven by mother age at marriage, maternal nutrition status, and prenatal visits to the hospital. The improvement in weight-for-height *z*-score and weight-for-age *z*-score were highly significant, and the covariates explained this change by 18 percent and 50 percent in sequence. Among the total explained portion, the major contribution is due to the better nutritional status of mothers and the wealthy status of households. In Sindh, the major drivers of progress in height-for-age *z*-score are similar to those in Punjab: maternal age at marriage, mother body mass index, prenatal visits, and wealth index, but none of these were significant. For the other two measures of nutrition, the change observed in the past five years was positive but not significant. The largest improvement has been found for the weight-for-age *z*-score compared to the other two indicators, and the changing factors found to be the mother’s own nutritional status, visits to the hospital before pregnancy, and household assets.

Balochistan has shown a notable reduction in stunting among children, as it has declined by 35 percent, the largest improvement compared to other regions of Pakistan. Although no predictor was significantly contributing to the change, the coefficients of mother age at marriage, prenatal visits to health facilities, and wealth index were positive. Surprisingly, the coefficient of mother employment in the Balochistan model was positive and the highest compared to other regional models. The economic condition of households living in Balochistan also seems to have improved in the last five years since the coefficient of the wealth index is almost double as compared to other regions of Pakistan.

Gilgit Baltistan has shown mixed findings where the height-for-age *z*-score and weight-for-height *z*-score are slightly moved toward the stunted and wasted cut points, while the weight-for-age *z*-score has remarkably improved to almost become normal for all children in the sample. The change in height-for-age *z*-score is partially explained by the poor nutritional status of the mother and her not visiting the hospital before pregnancy. It suggests that the factors that contribute to positive changes in nutritional status in other regions are the same factors that are causing negative changes in Gilgit. The situation of children’s nutritional status living in ICT is better compared to all other regions, even five years ago. The highest improvement among all three indicators is in the weight-for-height *z*-score, but no statistically significant relationship was observed. The availability of safe drinking water, the wealth index, and prenatal visits were found to be positive drivers in our study.

#### 3.3.2. Robust Analysis

We conducted some additional analysis following previous studies to confirm whether the results were robust or not. First of all, children’s age is a critical factor in determining their nutritional outcome, and the first 1000 days are considered the most favorable time period for a child’s normal growth. In addition, some determinants, like the wealth index and parental education, may have higher determining power for older children. On the other hand, some factors that require recall memory, like prenatal visits, may have a strong association with young children. Results after dividing the sample into younger (less than and equal to 24 months) and older children (more than 24 months) confirmed our initial concerns. The coefficient value of mother education and wealth index was higher for older children, while prenatal visits were significant only for younger children. Secondly, keeping in mind the possible endogenous effect of education on health facility visits, we excluded the later variable, and the results indicated that the contribution of mother education increased in improving nutritional growth. This suggests that the impact of a mother’s education on a child’s health is partly mediated through access to health care facilities. Overall, with slight changes in the parameters, our results remain consistent.

## 4. Discussion

This paper investigates the trends in child nutritional status and analyzes the key drivers responsible for the changes in their nutritional status over a five-year period in Pakistan by employing nationally representative data sets. Results obtained from regression decomposition analysis highlighted that there has been a significant improvement in child undernutrition. The estimated coefficients show that the factors used in the model explain a pronounced reduction in child undernutrition. In line with existing studies, we found that maternal nutritional status and household asset accumulation contributed impressively to improving a child’s growth.

Mothers’ nutritional status has been documented numerous times as a crucial factor in a child’s normal growth [9,20]. One study from Pakistan also found that maternal health was significantly associated with stunting and wasting [22]. Similarly, Egyptian scholars concluded that a mother’s nutrition is a critical determinant that indicates how genetics and phenotype can influence the stature of a child [20]. The positive intergenerational effect of the mother’s health can also be witnessed in the success story of Sub-Saharan Africa. Researchers registered that maternity care and the provision of iron supplements have been effective channels to improve the child malnutrition situation in seven African countries [23]. In addition to the positive influence of a higher BMI on child nutrition, it is worthwhile to note that a BMI over 25 could also be detrimental to the mother’s own health in the long term and to later generations through epigenetic expression.

Economic status, a crucial factor, has also been consistently linked with child welfare. Intuitively, this means that a child residing in a wealthy household is more likely to have better access to nutritiously rich food than a child living with limited resources. A household that falls into the low-income category becomes a more vulnerable place for a child to receive proper nutrition [24]. We found that household economic status, measured as accumulated wealth in the form of assets, is improving all indicators of child nutrition in our sample data. On similar lines, a study from Ethiopia reported that low family income is a critical risk factor for acute malnutrition in children under the age of five [25].

Mother age at marriage was seen to play a critical role in improving child growth in this study since early marriages are associated with pregnancy at a young age. Adolescent pregnancy increases the risk of a low-birth-weight child because the mother herself is in the growth stage; the risk becomes even greater if her BMI is less than 18.5 [26]. Another indirect benefit of marrying late is better utilization of time in the form of acquiring skills or receiving a formal education, both of which can expand the earning source for a family. Our results are in accordance with previous literature; one study from Pakistan found that the likelihood of stunted growth for a child increases if a woman marries before 18 years of age [9].

Parents education level has increased over the past five years, and its effect on driving child nutritional scores toward better is also prominent, especially for mothers with higher education. This positive link can be attributed to multiple pathways, one of which is the delay and reduction in overall fertility. Some previous studies also highlighted that women with higher education are more likely to use contraceptives and have higher chances of delaying pregnancy [27]. Secondly, an educated mother has more chances to earn money, which she can spend on food and health-related expenses for the child [28]. Thirdly, education increases the overall capabilities of the woman within the household, and thus she would be more knowledgeable to divert resources toward the better health of the child [29]. Among all of the previous success stories in overcoming child malnutrition challenges, women’s education has been a significant factor. Studies from Bangladesh have reported the positive role of maternal education in elevating the child ‘s and mother’s health status in the past decade [19,30,31].

We found another critical factor to be consistently improving the nutritional score of children across different regions of Pakistan: prenatal visits to the hospital. Access to health care facilities is a prominent entry point to providing education on health and nutrition for the wellbeing of mother and child. Health providers can communicate effectively with parents on disease prevention, early diagnosis, and breastfeeding behaviors. Existing studies have also highlighted that families who receive antenatal care services are more likely to prefer institutional delivery and utilization of postnatal care [32]. Factors like father education and household environment were positive but showed a modest contribution.

Based on the incoherent growth of nutrition across different regions, we conducted separate decomposition analyses and found that the drivers of this change are also inconsistent. It provides an opportunity to advance research with more in-depth interviews in each region separately. For example, the predictors we used in our analysis could not explain the impressive growth of child nutrition in Balochistan. Future researchers may need to identify some new determinants in that region to explain the change in nutritional indicators.

Another exceptional result from regional analysis is the positive role of mother employment in improving all indicators of nutrition in GB and Balochistan. The role of a mother’s employment has been documented as one of the most contradictory factors [33]. On the one hand, the rise in a mother’s financial status may lead to better provision of food and health-related inputs, while on the flip side, there is less availability of care time for proper child health. It can be assumed that there may be some additional support (in terms of taking care of the child by some other family member in the absence of the mother) provided by the community for working mothers in GB and Balochistan. Another reason may be the type of employment in these areas; for instance, more females are involved in managerial, professional, or skilled jobs in the above-mentioned regions than in Punjab, where, along with professional jobs, women are also employed in services and unskilled manual jobs. All in all, we suggest a more detailed study specifically focusing on the link between mother employment and child nutrition, which can provide more insights.

## 5. Conclusions

Contrary to the continuous surge in child undernutrition, recently we started to observe a reverse trend, which became the motivation for this study. We aim to highlight essential contributing factors to this positive change by using the latest nationally representative datasets from Pakistan. Estimates from decomposition analysis confirmed the largely discussed determinants in existing studies, ranging from mother education, nutrition, age at marriage, and access to health facilities to household economic status, as key drivers of nutritional change. The findings from this study further reiterate the government’s efforts to expand community-based lady health workers since access to medical facilities is driving significant changes in the long-term nutritional status of children. The results also suggest that instead of designing policies considering common factors of nutrition in Pakistan, provincial governments may require resources to find out the distinct factors in specific areas. Taken together, there is no consolidated approach to dealing with child undernutrition; instead, it is a multifaceted problem that requires collaborative efforts between the education, health, and poverty alleviation sectors.

## Figures and Tables

**Table 1:** Changes in the mean HAZ, WHZ, and WAZ scores from 2012/2013 to 2017/2018.

	Punjab	Sindh	KPK	Balochistan	GB	ICT	Total
Mean HAZ scores							
2012-13	−1.58	−2.23	−1.61	−3.58	−1.15	−0.87	−1.77
2017-18	−1.33	−1.99	−1.52	−1.59	−1.81	−0.99	−1.54
Change	0.25	0.24	0.09	1.99	0.66	0.12	0.23
Percent change	−15.8	−10.8	−5.6	−55.6	57.4	13.8	−12.9
Mean WHZ scores							
2012-13	−0.53	−0.66	−0.34	0.65	0.33	−0.57	−0.51
2017-18	−0.18	−0.62	−0.19	−0.61	0.46	−0.02	−0.25
Change	0.35	0.04	0.15	1.26	0.13	0.55	0.26
Percent change	−66.04	−6.1	−44.1	193.9	−39.4	−96.5	−50.9
Mean WAZ scores							
2012-13	−1.28	−1.77	−1.18	−1.69	−0.46	−0.88	−1.38
2017-18	−0.88	−1.72	−1.02	−1.55	−0.74	−0.57	−1.1
Change	0.4	0.05	0.16	0.14	0.28	0.31	0.28
Percent change	−31.3	−2.8	−13.6	−8.3	60.9	−35.2	−20.3

Source: Authors’ calculations based on PDHS 2012-13, 2017-18.

**Table 2:** Changes in the mean/percentage of key variables from 2012-13 to 2017-18.

	Mean/Percentage 2012–2013	Mean/Percentage 2017–2018	Change	Percent Change
Maternal Age at Marriage	18	19	1	5
Maternal Body mass index (kg/m^2^)				
<18.5 (underweight)	14	10	−4	29
18.5–24.9 (normal)	53	46	−7	13
≥25 (overweight)	33	44	11	33
Prenatal Visits				
No	25	13	−12	48
Less than 4 visit	38	37	−1	3
4 and more visit	38	50	12	32
Delivery at hospital	49	67	18	37
Maternal Education				
No Education (years)	55	47	−8	15
Primary	17	16	−1	6
Middle	8	9	1	13
Secondary	15	19	4	27
Tertiary and above	5	9	4	80
Wealth Status of Household				
Poor	38	29	−9	24
Middle	33	33	0	0
Rich	28	37	9	32
Father Education (years)	6	7	1	17
Safe drinking water	93	92	−1	1
Hygienic sanitation	68	80	12	18

Source: Authors’ calculations based on PDHS 2012-13, 2017-18, using sampling weights.

**Table 3:** Linear regression decomposition estimates of child undernutrition in Pakistan.

	HAZ	%	WHZ	%	WAZ	%
2012/2013	−1.648 ***		−0.604 ***		−1.381 ***	
	−0.0529		−0.0459		−0.0439	
2017/2018	−1.421 ***		−0.298 ***		−1.066 ***	
	−0.0516		−0.037		−0.0436	
Total difference	0.227 ***		0.306 ***		0.315 ***	
	−0.0749		−0.0589		−0.0626	
Explained	0.183 ***	81	0.117 ***	38	0.173 ***	55
	−0.0495		−0.031		−0.0437	
Unexplained	0.0435	19	0.189 ***	62	0.143 ***	45
	−0.066		−0.0544		−0.0505	
Explained difference						
Maternal age at marriage	0.018 *	8	0.005	1	0.007	2
	−0.0097		−0.00424		−0.0047	
Maternal body mass index (≥25)	0.046 ***	20	0.036 ***	12	0.052 ***	17
	−0.0167		−0.0127		−0.0157	
Maternal Education						
Secondary	0.008	3	0.002	0	0.01 *	3
	−0.0068		−0.0046		−0.0056	
Tertiary and above	0.006	2	0.011 *	3	0.012 *	4
	−0.0064		−0.0063		−0.0068	
4 and more prenatal visits	0.047 ***	21	−0.00752	−2	0.015	5
	−0.0175		−0.0126		−0.0129	
Father Education (years)	0.004	2	0.008	2	0.008	3
	−0.0051		−0.0056		−0.0055	
Household wealth status	0.037 *	16	0.026 *	8	0.038 **	12
	−0.0193		−0.0142		−0.0177	
Region						
Punjab	−0.0008	0	0.0237 **	8	0.0097	3
	−0.0088		−0.0105		−0.0066	
Sindh	0.0006	0	−0.0004	0	−0.0009	0
	−0.0146		−0.0096		−0.0168	
KPK	0.005	2	−0.0003	0	0.0038	1
	−0.0057		−0.0019		−0.0043	
Balochistan	−0.003	−1	−0.0008	0	−0.007 **	−2
	−0.0056		−0.0039		−0.0036	
GB	0.014	6	0.0538 ***	17	0.0476 ***	15
	−0.0088		−0.0156		−0.0135	
ICT	0.0001	0	−0.0002	0	0.000001	0
	−0.0002		−0.0001		0.00009	
N	7494		7492		7499	

Source: Authors’ calculations based on PDHS 2012-13, 2017-18, using sampling weights. Robust standard errors in parentheses, *** *p* < 0.01, ** *p* < 0.05, * *p* < 0.10.

**Table 4:** Non-linear regression decomposition estimates of child undernutrition in Pakistan.

	Stunting	%	Wasting	%	Underweight	%
2012/2013	0.426 ***		0.132 ***		0.301 ***	
	−0.0155		−0.0143		−0.0149	
2017/2018	0.354 ***		0.0784 ***		0.217 ***	
	−0.0166		−0.008		−0.0131	
Total difference	0.0717 ***		0.0541 ***		0.0832 ***	
	−0.0228		−0.0164		−0.0199	
Explained	0.0381 ***	53	0.0102	19	0.0332 **	40
	−0.0136		−0.0093		−0.0131	
Unexplained	0.0336	47	0.0439 ***	82	0.0500 ***	60
	−0.0214		−0.0127		−0.017	
Explained difference						
Maternal age at marriage	0.003	4	−0.00008	0	0.0008	1
	−0.0022		−0.00058		−0.0013	
Maternal body mass index (≥25)	0.011 **	14	0.004	8	0.011 ***	13
	−0.00502		−0.0029		−0.0042	
Maternal Education						
Secondary	0.004 *	6	0.0013	2	0.005 **	6
	−0.0025		−0.0011		−0.0025	
Tertiary and above	0.005 *	7	0.002	3	0.004	4
	−0.0029		−0.0013		−0.0024	
4 and more prenatal visits	0.012 **	17	0.00008	0	0.0016	2
	−0.0052		−0.002		−0.004	
Father Education (years)	0.002	2	0.0003	1	0.002	3
	−0.0016		−0.0006		−0.0016	
Household wealth status	0.009 *	12	0.003	4	0.011 *	13
	−0.0051		−0.0025		−0.0057	
Region						
Punjab	−0.005	−7	0.0002	0	−0.0006	0
	−0.003		−0.0014		−0.0023	
Sindh	0.0001	0	−0.00003	0	−0.0002	0
	−0.0026		−0.00068		−0.0039	
KPK	0.001	1	−0.0002	0	0.0006	1
	−0.0012		−0.0003		−0.0008	
Balochistan	−0.002 **	−3	−0.001 **	−3	−0.002 **	−3
	−0.00105		−0.000673		−0.00104	
GB	0.0007	1	0.008 *	15	0.009 ***	12
	−0.0022		−0.0045		−0.00327	
ICT	0.00005	0	−0.00002	0	0.00003	0
	−0.00006		−0.000027		−0.000046	
N	7494		7492		7499	

Source: Authors’ calculations based on PDHS 2012-13, 2017-18, using sampling weights. Robust standard errors in parentheses, *** *p* < 0.01, ** *p* < 0.05, * *p* < 0.10.

## Data Availability

The datasets are available online and can be accessed through the DHS website https://dhsprogram.com/data (accessed on 10 July 2023).

## Supplementary Materials

The following are available online at https://www.mdpi.com/article/10.3390/nu15143124/s1, Table S1: Linear decomposition estimates of stunted child in different regions of Pakistan; Table S2: Linear decomposition estimates of wasted child in different regions of Pakistan; Table S3: Linear decomposition estimates of underweight child in different regions of Pakistan.
