# Supplementary material for: Drivers of Nutritional Change in Pakistan: A Decomposition Analysis
Source: Nutrients. 2023 Jul 13;15(14):3124. doi: 10.3390/nu15143124 (PMC10384884; doi:10.3390/nu15143124)
Supplement: Supplementary file 1 [file nutrients-15-03124-s001.zip › nutrients-2494471-supplementary.pdf]

nutrients

MDPI

Supplementary Material

Drivers of Nutritional Change in Pakistan: A Decomposition Analysis

Azka Rehman [1,2]*, Ping Qing [2] and Xia Cui [1]*

1 School of Economics and Statistics, Guangzhou University, Guangzhou, China; azkarehman@outlook.com
2 College of Economics and Management, Huazhong Agricultural University, Wuhan, China
* Correspondence: azkarehman@gzhu.edu.cn; cuixia@gzhu.edu.cn

Table S1: Linear decomposition estimates of stunted child in different regions of Pakistan

	Punjab	Sindh	KPK	Balochistan	GB	ICT
2012/2013	-1.437***	-2.126***	-1.544***	-3.408***	-0.931***	-0.854***
	-0.070	-0.095	-0.119	-0.267	-0.268	-0.136
2017/2018	-1.279***	-1.801***	-1.301***	-1.282***	-1.480***	-0.993***
	-0.074	-0.111	-0.116	-0.219	-0.131	-0.149
Total Difference	0.158	0.325**	0.243	2.127***	-0.549*	-0.139
	-0.102	-0.147	-0.166	-0.345	-0.298	-0.202
Explained	0.267***	0.129*	0.169*	0.291	0.372**	-0.068
	-0.067	-0.072	-0.099	-0.204	-0.170	-0.111
Unexplained	-0.109	0.196	0.073	1.835***	-0.922***	-0.071
	-0.087	-0.139	-0.149	-0.333	-0.294	-0.214
Explained difference						
Maternal age at marriage	0.0316*	0.022	-0.005	0.084	0.073	-0.007
	-0.018	-0.017	-0.010	-0.070	-0.060	-0.017
Maternal body mass index (≥25)	0.0715**	0.019	0.078	0.019	-0.347*	0.001
	-0.031	-0.021	-0.053	-0.035	-0.186	-0.046

Maternal Education						
Secondary	0.017	0.007	-0.001	0.005	0.013	-0.011
	-0.013	-0.013	-0.005	-0.021	-0.028	-0.020
Tertiary and above	0.011	0.003	0.006	-0.002	-0.015	-0.002
	-0.013	-0.008	-0.010	-0.008	-0.042	-0.034
4 and more prenatal visits	0.0938**	0.049	0.048	0.012	-0.011	-0.087
	-0.039	-0.032	-0.035	-0.065	-0.027	-0.075
Father Education (years)	0.014	0.001	-0.005	0.002	0.028	-0.003
	-0.012	-0.009	-0.014	-0.013	-0.046	-0.040
Household wealth status	0.053	0.046	0.005	0.217	0.185	-0.012
	-0.035	-0.038	-0.026	-0.166	-0.124	-0.026
N	2054	1684	1491	1219	609	281

Source: Authors' calculations based on PDHS 2012-13, 2017-18, using sampling weights. Robust standard errors in parentheses, *** p<0.01, ** p<0.05, * p<0.1

Table S2: Linear decomposition estimates of wasted child in different regions of Pakistan

	Punjab	Sindh	KPK	Balochistan	GB	ICT
2012/2013	-0.667***	-0.714***	-0.350***	0.559**	0.245*	-0.621***
	-0.062	-0.089	-0.115	-0.263	-0.144	-0.133
2017/2018	-0.227***	-0.613***	-0.289***	-0.713***	0.386***	-0.040
	-0.049	-0.059	-0.093	-0.153	-0.090	-0.101
Total Difference	0.440***	0.102	0.061	-1.272***	0.141	0.580***
	-0.078	-0.106	-0.148	-0.304	-0.170	-0.167
Explained	0.0800*	0.055	0.049	-0.049	0.205*	0.078
	-0.046	-0.040	-0.076	-0.117	-0.107	-0.075
Unexplained	0.360***	0.047	0.012	-1.223***	-0.346*	0.502***
	-0.071	-0.103	-0.147	-0.307	-0.189	-0.156

Explained difference						
Maternal age at marriage	-0.004	0.003	0.008	0.001	0.0729**	0.003
	-0.011	-0.009	-0.015	-0.031	-0.034	-0.008
Maternal body mass index (≥25)	0.0324*	0.0536**	-0.020	0.033	0.101	0.041
	-0.020	-0.025	0.022	-0.053	-0.069	-0.043
Maternal Education						
Secondary	-0.002	-0.005	-0.000	-0.007	0.018	-0.014
	-0.010	-0.010	-0.002	-0.014	-0.027	-0.020
Tertiary and above	0.014	0.006	-0.000	0.001	-0.023	-0.002
	-0.011	-0.008	-0.005	-0.005	-0.029	-0.038
4 and more prenatal visits	-0.007	-0.019	-0.019	0.071	-0.004	0.028
	-0.026	-0.021	-0.028	-0.060	-0.018	-0.032
Father Education (years)	0.011	0.001	-0.005	-0.005	0.045	0.007
	-0.011	-0.004	-0.014	-0.024	-0.062	-0.030
Household wealth status	0.0852**	0.034	-0.017	-0.052	-0.201**	-0.001
	-0.037	-0.032	-0.025	-0.055	-0.086	-0.010
N	2054	1684	1491	1219	608	279

Source: Authors' calculations based on PDHS 2012-13, 2017-18, using sampling weights. Robust standard errors in parentheses, *** p<0.01, ** p<0.05, * p<0.1

Table S3: Linear decomposition estimates of underweight child in different regions of Pakistan

	Punjab	Sindh	KPK	Balochistan	GB	ICT
2012/2013	-1.288***	-1.763***	-1.156***	-1.625***	-0.389***	-0.907***
	-0.059	-0.076	-0.111	-0.215	-0.146	-0.117
2017/2018	-0.878***	-1.607***	-0.948***	-1.478***	-0.576***	-0.574***
	-0.061	-0.082	-0.092	-0.115	-0.090	-0.104

Total Difference	0.410***	0.156	0.208	0.147	-0.187	0.334**
	-0.085	-0.109	-0.144	-0.242	-0.172	-0.156
Explained	0.211***	0.090	0.121	0.042	0.073	0.005
	-0.062	-0.056	-0.087	-0.111	-0.134	-0.081
Unexplained	0.200***	0.066	0.087	0.106	-0.260	0.329**
	-0.068	-0.102	-0.132	-0.242	-0.190	-0.141
Explained difference						
Maternal age at marriage	0.014	0.009	0.002	0.052	-0.003	0.001
	-0.012	-0.010	-0.005	-0.043	-0.026	-0.005
Maternal body mass index (≥25)	0.0609**	0.0557**	0.060	0.026	-0.124	0.018
	-0.025	-0.026	-0.041	-0.036	-0.100	-0.032
Maternal Education						
Secondary	0.011	0.004	-0.001	-0.008	0.023	-0.007
	-0.010	-0.010	-0.003	-0.015	-0.027	-0.022
Tertiary and above	0.020	0.004	0.003	-0.001	-0.018	-0.000
	-0.013	-0.006	-0.005	-0.003	-0.030	-0.043
4 and more prenatal visits	0.047	-0.002	0.014	0.023	-0.016	-0.022
	-0.029	-0.021	-0.028	-0.038	-0.034	-0.028
Father Education (years)	0.016	0.003	-0.009	-0.002	0.047	-0.007
	-0.011	-0.006	-0.020	-0.011	-0.064	-0.024
Household wealth status	0.0872**	0.054	-0.011	0.079	-0.031	-0.004
	-0.037	-0.042	-0.020	-0.068	-0.063	-0.012
N	2054	1684	1491	1219	609	286

Source: Authors' calculations based on PDHS 2012-13, 2017-18, using sampling weights. Robust standard errors in parentheses, *** p<0.01, ** p<0.05, * p<0.1
